# Extranodal NK/T-Cell Lymphomas: The Role of Natural Killer Cells and EBV in Lymphomagenesis

**DOI:** 10.3390/ijms21041501

**Published:** 2020-02-22

**Authors:** Atif Saleem, Yasodha Natkunam

**Affiliations:** Department of Pathology, Stanford University School of Medicine, Stanford, CA 94305, USA; asaleem@stanford.edu

**Keywords:** NK cells, extranodal NK/T-cell lymphoma, EBV

## Abstract

Natural killer (NK) cells are lymphocytes involved in innate and adaptive immune functions. They are the presumed cell of origin of distinct hematolymphoid malignancies, including aggressive NK-cell leukemia and extranodal NK/T-cell lymphoma (ENKTL). This review focuses on the role of NK cells and Epstein–Barr virus (EBV) in ENKTL pathogenesis.

## 1. Introduction to NK-Cell-Derived Hematolymphoid Malignancies

Natural killer (NK) cells are lymphocytes with innate and adaptive immune characteristics, which were first observed in 1969 and officially defined in 1975 [1]. These cells comprise approximately 2%–20% of peripheral blood lymphocytes and exhibit a myriad of different functions, including the ability to lyse infected cells, initiate recruitment of additional immune components, generate antigenic memory responses, and survey and destroy neoplastic cells. As the name may imply, these cells are able to “kill” tumor and virus-infected cells without any predicated major histocompatibility complex (MHC) restriction. Inhibitory NK-cell receptors include killer-cell immunoglobulin-like receptors (KIRs) which bind to MHC class I molecules, the Ly49 family of receptors which bind to MHC class I molecules, and the inhibitory NK cell receptor, NKG2A, which recognizes non-classical MHC molecules [2]. Tumor cells and virus-infected cells lose MHC class I (ordinarily present on all healthy cells) expression which demises the capability of the KIRs on NK cells to bind and propagate an inhibitory signal [2]. This, however, is coupled with the upregulation of activating ligands in these aberrant cells which ultimately bind to and activate NK cells. NK cells can be activated to destroy cells that are “missing self”. The “missing self” hypothesis proposed by Dr. Klas Kärre in 1981 states that NK cells can recognize abhorrent cells by detecting missing information in the target cell which is present in the healthy host cells; the “self” corresponding to MHC class I expression [3]. Activating NK-cell receptors include Ly49D/Ly49H, NKG2D, and natural cytotoxicity receptors and though CD16, which is a transmembrane receptor responsible for mediating antibody-dependent cellular cytotoxicity, can independently trigger NK cell degranulation, there must be synergistic co-activation of a combination of the aforementioned receptors to instigate this phenomenon. Cells which lack MHC class I expression can, in conjunction with the detection of stress-induced molecules, activate NK cells by signaling through immunoreceptor tyrosine-based activation motifs (ITAMs) [4], ultimately resulting in calcium mobilization followed by secretion of lytic granule contents onto the target cell and inevitable cell death. 

NK-cell degranulation is the process by which cytotoxic proteins such as perforin and granzyme are cytoplasmically released, and this is a rapid form of cellular death as contrasted to antibody-dependent cellular cytotoxicity (ADCC) in which NK-cell surface CD16 binds to the Fc portion of a bound immunoglobulin, resulting in activation of the NK cell and ultimate cell death. In an effort to control early viral infections given the increased time it takes for the adaptive immune response to engage, NK cells also produce and secrete cytokines and chemokines such as IFN-gamma, tumor necrosis factor, IL-5, and IL-1 [5]. 

NK cells are the presumed cell of origin for multiple hematolymphoid malignancies, including aggressive NK-cell leukemia and extranodal NK/T-cell lymphoma (ENKTL). This review focuses on the role of NK cells in the lymphomagenesis of ENKTL, and it’s the interplay with Epstein–Barr virus (EBV), a defining feature of this neoplasm. The role of NK cells in tumor cell surveillance is well elucidated [1]. There is, however, a paucity of data characterizing the pathogenesis of NK cell proliferation and NK-cell-derived malignancies. Key reasons for this lack of data relate to their geographic distribution (they are uncommon except in East Asian and Central and South American populations), their variable association with EBV, and the logistical difficulty in studying viable tumor cells since ENKTL is often associated with extensive necrosis and inflammation. 

In the current World Health Organization (WHO) classification, a spectrum of indolent, and aggressive lymphoproliferative disorders (LPDs) arising from NK cells are recognized [6]. Chronic LPDs of NK cells include chronic active EBV infection (CAEBV) of T- and NK-cell type, which may have cutaneous as well as systemic manifestations, including hydroa vacciniforme-like lymphoproliferative disorder and severe mosquito bite allergy. Aggressive malignancies arising from NK cells include EBV-positive T- and NK-cell lymphomas of childhood, ENKTL, and aggressive NK-cell leukemia.

The common etiological association in these diseases is a presumed viral activation of EBV instigating aberrant NK-cell proliferation. There is a stark juxtaposition in the prognosis of NK-derived LPDs with or without EBV, with those associated with EBV demonstrating a more aggressive clinical course. Some NK LPDs are associated with hemophagocytic lymphohistiocytosis (HLH), which can precipitate a fulminant clinical course with rapid dissemination, multi-organ failure, and death [6]. 

ENKTL is the fifth most frequent peripheral T-cell lymphoma, which almost exclusively occurs in non-nodal sites; over 80% of cases occur in the nose, nasopharynx, and oropharyngeal regions [7]. Of note, when considering Asian, Central, and South American populations, ENKTL becomes one of the most common lymphomas arising from T- and NK-cells. The median age at diagnosis is 52 years, and there is a male preponderance (male-to-female ratio of roughly 2:1) [6]. On histopathologic examination, ENKTL is characterized by ulceration of involved mucosal sites with a diffuse, permeative lymphomatous infiltrate composed of variably sized tumor cells in a background of confluent necrosis and vascular damage [6]. At our institution the prototypical cocktail of ancillary studies always includes an in situ hybridization to detect EBV-encoded small RNAs (EBER), which are noncoding RNAs ubiquitously expressed in nuclei of EBV-infected cells and immunohistochemistry for CD56, CD4, and CD8 to aid in determining lineage and cytotoxic granule-associated markers, TIA-1, granzyme, and perforin. Although several of these immunohistochemical markers are common to T-cell subsets and T- lineage LPDs, the constellation of CD56, EBER, and cytotoxic markers, together with the clinical presentation provides excellent specificity for the diagnosis of ENKTL. 

## 2. The Cell of Origin in ENKTL: NK Cells or T-Cells?

Ambiguity regarding the cell of origin of ENKTL of either an NK or T-cell is reflected in its name. Various studies have described rates attributing 75%–90% of cases to be of NK lineage contrasted with approximately 10%–25% of T-cell lineage. A study of 67 ENKTL from Thailand demonstrated that upper aerodigestive tract ENKTL of T-cell origin compared with those of NK-cell origin showed a trend for better survival [8]. In this study, ENKTLs of NK-cell lineage (as compared to T-cell lineage) demonstrated more frequent expression of CXCL13 (59% vs. 0%), CD56 (83% vs. 33%), Oct-2 (38% vs. 0%), and IRF4/MUM1 (54% vs. 20%); PD-1 expression was less frequent in NK-cell-derived ENKTL (0% vs. 40%) as compared to those of T-cell lineage. Lack of expression of αβ and γδ T-cell receptor proteins in conjunction with the absence of a T-cell receptor gene rearrangement also suggests NK- cell lineage. Certain markers, such as granulysin, a saposin-like protein that functions as a pro-inflammatory and cytolytic molecule expressed by cytotoxic T-cells and NK cells, are expressed in NK cells and NK-cell-derived neoplasms. Granulysin-expressing cells often co-expressed CD56, whereas most CD4- and CD8-positive T-cells did not express granulysin in a study by Bello et al. [9] and can at times be the only positive marker when other cytotoxic markers are negative. 

## 3. The Role of NK Cells in Lymphomagenesis

EBV is a double-stranded DNA-containing human herpes virus, namely HHV-4 [10]. EBV typically infects B-cells through CD21 receptors, which bind to the glycoproteins gp350/220 on EBV. Less commonly, T-cells, epithelial cells, monocytes, and NK cells may also be infected by EBV. EBV infected NK cells secrete IL-2 and IL-9, which are known to be involved in NK-cell activation and proliferation. Following binding, there is endocytosis of EBV into the cellular cytoplasm, which subsequently undergoes replication of the EBV genome and ultimately resides in an episomal form. EBV demonstrates three different latency patterns, 1, 2, and 3, where there is differential expression of various viral proteins, which play a role in its virulence [11]. A type II latency pattern is most commonly seen in ENKTL which is characterized by expression of EBNA1 and LMP1 viral proteins.

EBV has been shown to contribute to oncogenesis in other hematologic neoplasms such as Burkitt lymphoma, in which the virus plays a complementary role in the overexpression of c-Myc and TCF3, exerting an anti-apoptotic function. This is in contrast to EBV-positive Hodgkin lymphoma, diffuse large B cell lymphoma, and ENKTL in which there is constitutive expression of the viral protein LMP1 which leads to activation of the protein complex NF-κB and Janus kinase/signal transducer and activator of transcription (JAK/STAT) pathway signaling. In nasopharyngeal carcinoma, however, NF-κB activation and global hypermethylation are thought to be facilitated by LMP1 [12]. 

In ENKTL, the tumor most commonly occurs at sites of primary EBV infection, and one hypothesis for this finding is that the initial EBV infection occurs while NK cells attempt to lyse an EBV infected cell [12]. Several other mechanisms by which EBV infects T-/NK cells have been proposed, including through using integrins as a receptor. Given the evidence of “dual infections” in both T-cell and NK-cell lineages in patients with CAEBV, one hypothesis suggests that EBV infects a common precursor cell. An additional theory suggests that immunological synapses facilitate virus transfer from an infected cell to T- or NK cells [13]. 

### 3.1. Presumed Pathogenetic Mechanisms Describing ENKTL Oncogenesis 

**Figure 1:**
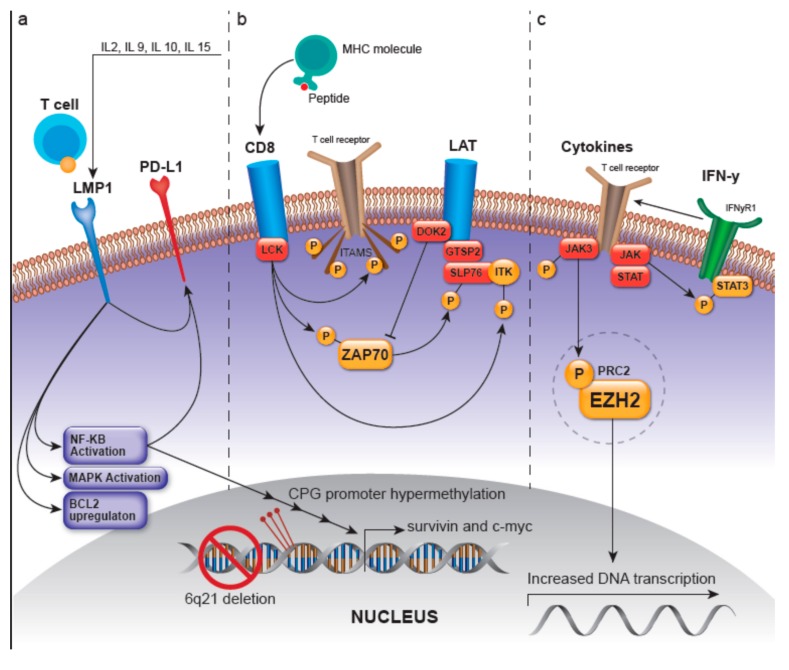
Pathogenetic mechanisms in extranodal NK/T-cell lymphoma (ENKTL). (**a**) LMP-1, a member of the TNF receptor family, is constitutively activated and leads to ligand-independent activation of NF-kB and mitogen-activated protein kinase (MAPK). LMP1/EBNA2 viral proteins transcriptionally target c-myc and activate NF-kB, which translocates into the nucleus and regulates MYC to induce proliferation and activate survivin. They also increase the sensitivity of the infected cell to IL-2, which promotes growth. LMP-1 upregulates PD-L1, which is postulated to occur through the MAPK/NF-kB pathway. (**b**) LCK/ZAP70: the kinase LCK is activated after major histocompatibility complex (MHC)-peptide binding, or presumably by genetic alteration, and can then phosphorylate immunoreceptor tyrosine-based activation motifs (ITAMs), leading to the recruitment of the protein tyrosine kinase ZAP70. Lymphocyte-cell-specific protein-tyrosine kinase (LCK) activation can also lead to autophosphorylation of ZAP70, which in turn activates the LAT and SLP-76 and the PLC-γ and MEK/extracellular signal-regulated kinase (ERK) pathways. The recruitment and activation of LAT and its subsequent recruitment of several molecules such as GRAP2 and ITK, modulate T-cell transcriptional regulation and gene expression related to survival, apoptosis, proliferation, and migration of T-cells. Docking protein 2 (DOK2) can impart negative feedback on NK-cell activation with presumed inhibition of ZAP-70. LCK activation also leads to the phosphorylation and activation of ITK. (**c**) JAK/STAT and EZH2: JAK/STAT represents a pro-proliferative signaling pathway, mutations in which can cause constitutive activation in ENKTL. JAK3 and STAT3 phosphorylation have been reported to occur in a majority of tested ENKTL cases and can also cause presumed activation of this pathway. Enhancer of zeste homolog 2 (EZH2) is a histone methyltransferase and part of the PRC2 complex and is involved in epigenetic regulation. Phosphorylation of EZH2 by JAK3 can initiate dissociation of PRC2 which leads to decreased H3K27me3 levels and confers EZH2 independent activity as a transcriptional coactivator for genes involved in DNA replication and tumor invasion. Abbreviations: LMP-1: latent membrane protein 1; TNF: tumor necrosis factor; NF-kB: Nuclear Factor kappa-light-chain-enhancer of activated B cells; IL-2: interleukin-2; PD-L1: programmed death ligand 1; ZAP70: zeta-chain associated protein kinase 70; JAK/STAT: Janus kinase/signal transducer and activator of transcription; LAT: linker for activation of T-cells; SLP-76: SH2 domain containing leukocyte protein of 76kDa; PLC-γ: phospholipase C-γ; MEK: mitogen-activated protein/extracellular signal-regulated kinase kinase; GRAP2: GRB2 related adaptor protein 2; ITK: inducible T cell kinase; EZH2: enhancer of zeste homolog 2; JAK3: Janus kinase 3; STAT3: signal transducer and activator of transcription 3; PRC2: polycomb repressive complex 2.

Presumed pathogenetic mechanisms describing ENKTL oncogenesis have primarily been based on canonical T-cell and NK-cell signaling pathways, three of which are summarized below and in Figure 1, including the 1) LMP-1-, 2) Lymphocyte-cell-specific protein-tyrosine kinase/ zeta-chain associated protein kinase 70 (LCK/ZAP70)-, and 3) JAK/STAT- and enhancer of zeste homolog 2 (EZH2) -involved pathways. However, environmental or epigenetic factors must occur in conjunction with genetic and epigenetic aberrancies in an ethnogenetically-predisposed individual to promulgate ENKTL lymphomagenesis.

#### 3.1.1. LMP-1-Related Pathways 

One postulated mechanism by which EBV infected NK cells progress to lymphoma is via the promotion, proliferation, and overexpression of LMP1, an oncogenic viral protein, induced by IL-2, IL-9, and IL-10. However, instead of LMP1 directly transforming and immortalizing cells during lymphomagenesis, it is postulated that this viral protein increases the sensitivity of the EBV-infected NK or T-cells to the proliferative effects of IL-2 [14]. 

An early model of ENKTL pathogenesis described after genome-wide gene expression profiling of 33 ENKTLs by Ng et al. [15] showed that a significant percentage of cases overexpressed c-Myc, p53, NF-κB, and p50 by immunohistochemistry with notable overexpression of survivin (97% of cases); survivin is a protein in the inhibitor of apoptosis (IAP) family which ultimately imparts an antiapoptotic effect through prevention of caspase-9 activation in the intrinsic apoptotic pathway [16]. It is theorized that after EBV infection, LMP1 and EBNA2 viral proteins transcriptionally target c-Myc and can activate NF-κB via the action of LMP1 mimicking a constitutively-active CD40-like receptor [17]. Upon ligand-independent activation of this receptor, the mitogen-activated protein kinase (MAPK) is activated and there is also nuclear translocation of NF-κB which then exerts its numerous functions, including the regulation of MYC to cause proliferation, or causing it independently, and transcriptionally activating survivin [18]. Following mutation and subsequent deregulation of the tumor suppressor p53, this pathway concomitantly up-regulates survivin [15]. 

LMP-1 also contributes to the upregulation of apoptosis-regulating genes such as BCL2 as well as the activation of additional pathways, including those that involve p38, MAPK/ERK1/2, JAK/STAT, ERK, MAPK, IRF4, PI3K/Akt, and Wnt signaling. LMP1, through the MAPK/NF-kB pathway, is also postulated to upregulate the cellular transmembrane protein programmed death-ligand 1 (PD-L1) which is crucial in suppressing the adaptive immune response and therefore contributing to tumor cell immune evasion [19]. 

#### 3.1.2. LCK/ZAP70-Related Pathways

As an overview, the first molecule recruited following MHC-peptide binding in the T-cell receptor (TCR) signaling pathway is LCK, which is crucial in the selection and maturation of developing T-cells. NK-gene expressional signature in ENKTLs has been shown to demonstrate higher LCK expression [20]. MHC-peptide binding or genetic alteration can activate LCK, which can then phosphorylate ITAMs, leading to the recruitment of the protein tyrosine kinase ZAP70 [21]. LCK activation can also lead to:

(1) Autophosphorylation of ZAP70, deeming it active and able to phosphorylate the linker for activation of T-cells (LAT) and **SH2 domain containing leukocyte protein of 76kDa** (SLP-76), where SLP-76 can subsequently activate the PLC-gamma pathway and the MEK/extracellular signal-regulated kinase (ERK) pathway [2,21]. LAT is a phosphoprotein that localizes to glycosphingolipid-enriched microdomains (GEMs) and acts as a docking site for proteins that contain an Src homology-2 (SH2) domain [22]. The LAT signalosome, which ultimately promotes T-cell signaling, is composed of LAT, and GRAP2/GADS (GRAP2 protein is involved in leukocyte-specific protein-tyrosine kinase signaling and contains an SH2 domain) [22]. The protein tyrosine kinase ZAP70 along with GRAP2/GADS have been shown to be expressed in greater than 90% and 68% of ENKTLs, respectively, with the GRAP2/GADS expression rate being higher in those ENKTLs of T lineage [23]. Moreover, GRAP2/GADS-positive ENKTLs frequently co-express docking protein 2 (DOK2). DOK2 protein may be involved in modulating BCR-ABL signaling and cellular proliferation induced by IL-4, 2, and 3 [22]; it is involved in negatively regulating GRAP2/GADS and NK-cell activation via inhibition of ZAP70 [23,24].
(2) Recruitment and activation of LAT which then recruits several molecules including GRAP2 and the tyrosine kinase IL-2 inducible T-cell kinase (ITK) to ultimately modulate T-cell transcriptional regulation and gene expression of survival, apoptosis, and proliferation-related genes as well as those related to the migration of T-cells [23].
(3) Phosphorylation and activation of ITK [25]

#### 3.1.3. JAK/STAT- and EZH2-Involved Pathways

The JAK/STAT pathway is up-regulated in ENKTL as compared to healthy NK cells and confers a pro-proliferative advantage. Mutations in this pathway can cause constitutive activation in ENKTL [26,27]. Moreover, Janus kinase 3 (JAK3) and signal transducer and activator of transcription 3 (STAT3) phosphorylation have been reported to occur in a majority of tested ENKTL cases and can also cause presumed activation of this pathway. The H3K27-specific histone methyltransferase, EZH2 is a part of the polycomb repressive complex 2 (PRC2), which isinvolved in epigenetic regulation. Overexpression of EZH2 has been shown to be associated with higher tumor cell proliferation, advanced stage, and predicted poorer overall survival [28]. An inverse relationship with H3K27me3 overexpression has also been described, which correlated with lower tumor cell proliferation, earlier stage, and was predictive of better overall survival. Phosphorylation of EZH2 by JAK3 can initiate dissociation of PRC2 which leads to decreased H3K27me3 levels and confers EZH2-independent activity as a transcriptional coactivator. This can lead to increased transcription of genes involved in cell cycle regulation such as cyclin D1, DNA replication, and tumor invasion.

#### 3.1.4. Other Ungrouped Pathways

Atypical NK cells in ENKTL have been described as having an undifferentiated immunophenotype similar to immature NK developmental stages, and their differentiation is shaped by epigenetic modifications. The role of epigenetics in ENKTL lymphomagenesis has recently been explored with microRNAs (miRNAs) frequently being shown to be dysregulated, for example, as promoter hypermethylation [27]. The most frequent chromosomal losses observed in studies of ENKTL are at 6q21 which leads to silencing of tumor suppressor genes such as *PRDM1, ATG5, AIM1, FOXO3,* and *HACE1* [29]. *PRDM1/BLIMP1* is a transcriptional repressor expressed by NK cells during development and is a negative regulator of NK function, showing tumor suppressor activity; in NK lymphoma, *PRDM1* silencing occurs via deletion of 6q21 and CpG promoter hypermethylation. The microRNA miR-223 mediates the downregulation of PRDM1 in ENKTL and evaluation of this tumor suppressor may offer a predictor of overall survival [20]. Similarly, expression of *FOXO3* (a member of the FoxO transcription factor family) and *PRDM1* has been shown to inhibit NK-cell proliferation in an experimental model [20,30,31]. The alteration of these transcription factors, PRDM1, and FOXO3, may impart a significant role in NK-cell lymphomagenesis and has the potential to offer insight for therapeutic targets.

Additionally, a hypothesis has been proposed in which EBV mediates miRNA deregulation by downregulation of the miRNAs let-7g, let-7a, and let-7c; EBV is also proposed to upregulate miR-155, which has presumed oncogenic function [32,33]. Epigenetic deregulation through mutations of BCL-6 corepressor (BCOR) and mixed lineage leukemia 2 (MLL2) have also been reported in ENKTL, as well as variations in other epigenetic modifiers such as ASXL3, ARID1A, and EP300 and are hypothesized to contribute to ENKTL pathogenesis [27]. 

A subset of ENKTL cases are preceded by a chronic inflammatory or lymphoproliferative disorder, namely CAEBV, hydroa vacciniforme-like lymphoproliferative disorder, and/or mosquito bite hypersensitivity. In contradistinction to CAEBV-derived cell lines, ENKTL cell lines are characterized by overexpression of genes related to growth factor activity, apoptosis, cell growth, signal transduction, and cell adhesion. It has been shown that LMP1 expression is induced and LMP1-inducible cytokine IP10 (chemoattractant which results in monocyte congregation) is secreted when monocytes transport IL-15 to EBV-positive NK/T-cells; this has been proposed as one theory that correlates these potential precursor lymphoproliferative disorders and their inflammatory nature with the progression to ENKTL [34]. 

## 4. Ethnogenetic Predisposition of ENKTL

A lack of data exists regarding any association between ethnicity and genetics in the predisposition to ENKTL. Li et al. described the results of their genome-wide association study (189 cases and 97 controls) of a population from the Guandong Province in southern China, suggesting that a common genetic variation, namely rs9277378 at the *HLA-DPB1* gene on chromosome 6 is a strong contributor to ENKTL [35]. This genetic variation changes the peptide-binding groove of HLA-DP, impairing antigen presentation, resulting in immune dysfunction and an inability to effectively clear EBV infection. Moreover, germline genetic variants in other EBV-associated malignancies such as nasopharyngeal carcinoma and a subset of Hodgkin lymphoma also showed strong associations with the MHC loci [35]. 

A vital feature of the hypothesis that an ethnogenetic predisposition may ineffectively clear EBV infections was described by Midgley et al. in a 2003 study where a relationship was mapped between HLA-A11 positive Chinese patients and poor recognition of EBV type 1 epitope variants [36]. In this study, 2 HLA-A11-restricted epitopes within the viral protein EBNA3B, namely IVT and AVF were analyzed and found to often be mutated in EBV strains in Papua New Guinea and southern China, both of which are areas where more than 50% of individuals carry the HLA-A11 allele. In this study, HLA-A11 positive Chinese patients demonstrated poor recognition of EBV type 1 epitope variants by IVT- or AVF-specific cytotoxic T-lymphocytes. It remains unclear whether recognition by an NK-cell line would display similar results [36]. 

In Central and South America, descriptive studies regarding ENKTL have been performed; however, no analysis of the ethnogenetic predisposition nor molecular sequencing of these tumors is available. This dearth is despite an increased frequency of ENKTL in those of Mayan descent, which raises a potential component of shared ancestry with East Asian populations with a similar genetic predisposition [37].

There are also factors such as germline genetic variations, which may provide insight on ethnogenetic predisposition, for example, a positive association between the HLA-A26 genotype and EBV-positive NK/T-cell lymphoproliferative disorders. Of note, the A26 alleles are frequently seen in East Asia, where the prevalence of this disease is high [38].

## 5. Therapeutic Strategies

The prognosis of ENKTL is generally poor: in the absence of treatment, overall survival is in the order of a few months. Most patients irrespective of the stage of disease are treated with a combined modality including chemotherapy and radiation. Another therapeutic approach is treatment with the SMILE (dexamethasone, methotrexate, ifosfamide, L-asparaginase, etoposide) regimen [39]. Moreover, though treatment with conventional chemotherapy or radiation therapy does appear to increase survival, other therapies, some still investigative, have been explored, including hematopoietic cell transplantation, immune check-point blockade/PD-1, and CD30- and CD38-targeted antibody blockade [40]. Patients with localized ENKTL predominantly undergo combined modality therapy with concurrent radiation therapy and chemotherapy, whereas patients with disseminated disease are suggested to receive a combination chemotherapy regimen including L-asparaginase [40]. Resistance to Adriamycin- and cyclophosphamide-based chemotherapy regimens are a well-described occurrence due to high expression of the *multidrug resistance (MDR)1/ABCB 1* gene and its product, P-glycoprotein [40,41]. L-asparaginase therapy is a frequent addition to combined modality therapy given the high sensitivity to this agent thought to be secondary to lack of asparagine synthase activity by NK cells [40]. Specific therapeutic strategies as they pertain to the various pathogenetic mechanisms in ENKTL are described next and summarized in Table 1. 

A recent study demonstrated that 3-deazaneplanocin A, an EZH2 inhibitor, was shown to suppress NK tumor cell growth and increase the apoptosis of these cells and treatment with a JAK3 inhibitor such as tofacitinib enhanced H3K27me3 in ENKTL tumor cell lines [28]. Tofacitinib decreased the expression of EZH2 and p-STAT3 with increased expression of H3K27me3 [42,43]. A selective JAK3 inhibitor PRN37 has been shown to cause more tumor growth inhibition than tofacitinib in an ENKTL JAK3 mutated xenograft model [44]. Stattic, a STAT3 inhibitor, has also been shown to induce apoptosis and inhibit viability in STAT3-mutated ENKTL cell lines [45].

Bortezomib, a proteasome inhibitor, was shown to inhibit ENKTL cells in vitro via a mitochondria-mediated caspase pathway, which inhibited the NF-κB pathway and ultimately induced apoptosis [46]. The anti-PD-1 inhibitor Pembrolizumab was studied as an investigative therapeutic agent in ENKTL to employ checkpoint inhibition given that PD-L1 has been shown to be up-regulated by LMP1, and demonstrated high efficacy [27,47]. Brentuximab vedotin, an antibody-drug conjugate targeting CD30, is thought to result in direct cytotoxicity of tumor cells and has been shown to contribute to the treatment of two refractory cases of ENKTL [48]. 

A recent study has demonstrated in vivo eradication of ENKTL by EBV-specific induced pluripotent stem-cell-derived antigen-specific cytotoxic T-cells, which remain as memory T-cells in the spleens of murine models for several months [49]. Moreover, LMP1/2a-specific cytotoxic T-cells have been shown to act as novel adoptive immunotherapies with successful outcomes in overall survival (100%) and progression-free survival (90%) in a study of 10 ENKTL patients who showed complete response to induction therapy [50]. Though clinical trials have not yet been established, inhibition of survivin by pharmaceuticals such as terameprocol have been shown to increase apoptosis and decrease tumor cell proliferation [15,51]. 

In a study by Go et al., dozens of miRNAs were shown to be down-regulated by methylation, and key miRNAs, including miR-34a and miR-181c were identified as potential therapeutic targets given their role in regulating PDGFRα, STAT3, and K-RAS [52]. Preliminary findings have shown that ENKTL cell lines were highly sensitive to the demethylating agent 5-azacytidine which adds to the prospectus of potential future therapies [42]. 

## 6. Conclusions

The pathogenesis of NK-cell-associated neoplasms remains elusive. In recent years, however, several lines of investigation have shed light on potential pathogenetic mechanisms and risk factors. The observation that certain germline genetic variants are strongly linked to ENKTL risk and cause an intrinsic impairment in the inability to clear EBV infection and well as the roles of *PRDM1/BLIMP1* and *FOXO3* in the induction of lymphomagenesis are of particular interest. Given the dismal survival of patients with ENKTL even with chemo- and/or radiation therapy, several potential therapeutic targets are under investigation, including PD-1, CD30, CD38, specific miRNAs, EZH2, and JAK3. Overall, more studies addressing genetic and epigenetic factors are needed, especially those that dissect different demographics and variations in clinical presentations and aggressivity among NK-cell-derived malignancies.

## Figures and Tables

**Table 1:** Emerging Therapeutic Possibilities in Extranodal NK/T-cell Lymphoma.

Pathway	Therapeutic	References
JAK/STAT	Tofacitinib (JAK inhibitor)/PRN371 (JAK3 selective inhibitor)/Stattic (STAT3 inhibitor)	[44,45,46]
EZH2	3-deazaneplanocin A (EZH2 inhibitor)	[29,44]
NF-κB	Bortezomib	[47]
PD-1	Pembrolizumab	[28,48]
CD30	Brentuximab	[49]
LMP-1	LMP1/2a-specific CTL	[51]
Survivin	Terameprocol	[15,52]
